# Apport des signes cutanés sur le diagnostic précoce d'une maladie périodique: à propos d'un cas

**DOI:** 10.11604/pamj.2019.33.16.18300

**Published:** 2019-05-10

**Authors:** Saer Diadie, Maïmouna Sow, Maodo Ndiaye, Boubacar Ahy Diatta, Diop Khadim, Suzanne Oumou Niang

**Affiliations:** 1 Dermatologie, CHU Aristide Le Dantec, Dakar, Sénégal; 2 Médecine Interne, CHU Aristide Le Dantec, Dakar, Sénégal

**Keywords:** Maladie périodique, peau, Dakar, Periodic disease, skin, Dakar

## Abstract

Les auteurs rapportent l'observation d'une maladie périodique diagnostiquée à l'aide de placards érysipélatoïdes récidivants et persistants sous antibiothérapie. L'interrogatoire, les tests génétiques et l'évolution favorable sous colchicine ont permis d'asseoir le diagnostic.

## Abstract (en)

We here report a case of chronic periodic disease diagnosed on the basis of recurrent and persistent erysipelas in plaques in a patient under antibiotic therapy. Patient's interview, genetic testing and favorable outcome of colchicine helped to reach a diagnosis.

## Introduction

La fièvre méditerranéenne familiale (FMF) ou maladie périodique (MP) est une fièvre récurrente héréditaire à transmission autosomique récessive associée à une mutation du gène MEFV. Elle touche les populations originaires du pourtour de la méditerranée [1]. Elle n'est pas décrite en Afrique subsaharienne. Les manifestations cliniques sont pléomorphes et aspécifiques d'où le retard diagnostique pouvant aller jusqu'à 10,75 ans [2]. L'atteinte cutanée est rare et peut être confondue à tout point de vue à un érysipèle [3]. Nous rapportons le premier cas de MP au Sénégal à révélation cutanée et confirmée par les tests génétiques.

## Patient et observation

Il s'agissait d'une patiente âgée de 29 ans, mauritanienne, de phototype V. Elle appartient à une famille d'origine saoudienne. Son tableau clinique a évolué 8 mois avant son admission marquée par une récurrence de fièvre avec arthralgies d'allure inflammatoire mal systématisées environ une poussée mensuelle. Ce tableau avait motivé des consultations avec recherche de maladie lupique et la polyarthrite rhumatoïde. Il n'existait pas d'arguments immunologiques notamment les anticorps anti DNA natifs, anti-Sm et anti-cyclic citrullinated peptide (anti CCP). Deux mois avant son hospitalisation, elle a présenté deux épisodes d'éruption cutanée à type de placard douloureux au niveau du dos qui avaient bien évolué après antibiothérapie à base d'amoxicilline à raison de 6g par jour pendant 7 jours. Suite à une récidive du même tableau clinique, la patiente est venue en consultation dans notre service.

**Figure 1:**
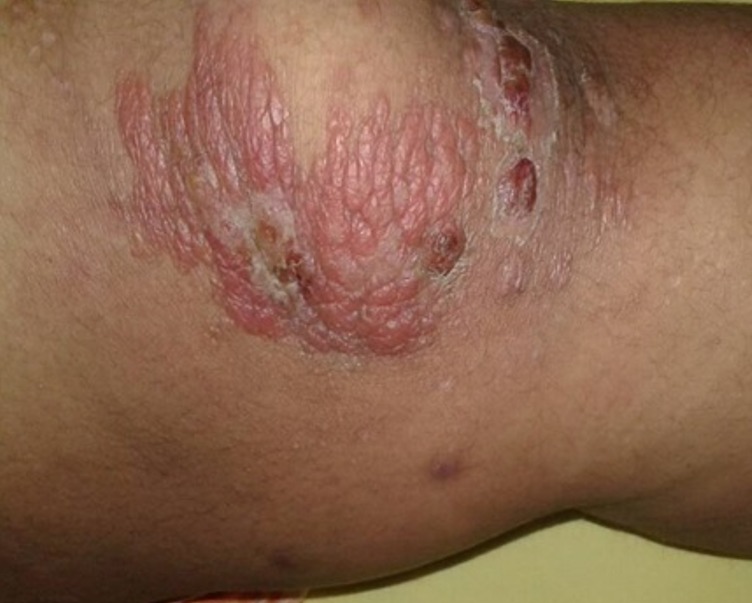
placard inflammatoire, aspect de pseudo érysipèle

A l'admission, l'état général était conservé. Il existait une fièvre à 39°C. L'examen dermatologique révélait des placards inflammatoires au niveau du dos et des membres inférieurs (Figure 1). On ne notait pas d'adénopathies. Les diagnostics d'érysipèle et de syndrome de Wells avaient été évoqués. A l'hémogramme on notait une hyperleucocytose à 19.000 éléménts/mm³ à prédominance neutrophilique 60% avec une thrombocytose à 600.000 éléments/mm^3^. La CRP (C réactive protéine) et la VS (vitesse de sédimentation) était positive respectivement à 146mg/l et 100mm à la première heure.

L'évolution était favorable suite au traitement par Amoxicilline-acide clavulanique à raison de 6g/j en intra veineuse pendant trois jours puis par voie orale. La suite était marquée par la réapparition du même tableau clinique alors que la patiente était encore sous antibiotique. Devant la récidive du tableau clinique, la maladie périodique avait été évoquée ainsi le génotypage réalisé avait mis en évidence une mutation du gène MEFV. La protéinurie était à 0,2g/24h. L'évolution était favorable sous colchicine (1mg/j) avec un recul de 5 mois suite auquel la patiente a été perdue de vue.

## Discussion

Nous avons rapporté l'observation d'une maladie périodique à révélation cutanée. Le diagnostic est retenu devant les antécédents de parenté saoudien, les épisodes de douleurs articulaires fébriles, la poussée de placard érysipélatoïde, la mise en évidence de la mutation du gène MEFV et l'évolution favorable sous colchicine.

La fièvre méditerranéenne familiale est une maladie auto-inflammatoire monogénique qui affecte les populations d'origine méditerranéenne [4]. Elle n'a jamais été décrite en Afrique subsaharienne. Elle est plutôt retrouvée au Maghreb [5]. La maladie périodique est une pathologie pédiatrique, survenant dans 80% des cas avant 20ans [2]. Elle se caractérise par une fièvre récurrente associée à une atteinte des séreuses et des articulations [6]. Les accès durent un à trois jours. La principale complication est l'amylose rénale qui est le facteur pronostic majeur [1]. Le risque de décès par l'amylose est corrélé à la précocité de début de la maladie [7].

Cette observation est particulière par le début tardif des symptômes et le court délai du diagnostic qui est de 8 mois. En effet, ce délai est en moyenne de 10,75 ans [2]. Ceci s'explique d'une part par la rareté de la maladie faisant qu'elle n'est pas évoquée à priori mais également de la non spécificité des manifestations cliniques qui erre le plus souvent le diagnostic. Notre patiente en est une parfaite illustration car initialement, les polyarthrites inaugurales avaient dérouté le diagnostic vers un lupus et une PR (polyarthrite rhumatoïde) mais les explorations immunologiques étaient en défaveur. Secondairement, la survenue de placard érysipélatoïde fébrile avec syndrome inflammatoire biologique avait de même faussement fait retenir un érysipèle. Le diagnostic a pu être redressé suite à la persistance du tableau cutané sous traitement antibiotique.

Le pseudo érysipèle malgré sa relative rareté (5% des cas) est une grande caractéristique de la maladie [3]. En effet, notre orientation diagnostique vient d'un cas de MP décrit précédemment dans notre service mais non rapporté qui s'est compliqué d'amylose au stade d'IRC (insuffisance rénale chronique). Il s'agit d'une patiente âgée de 24 ans suivie en néphrologie pour un syndrome néphrotique impur traité par corticothérapie dont la biopsie rénale avait conclu à une amylose AA. L'évolution était marquée par une survenue de récidive de placards érysipélatoïdes qui avait motivé son hospitalisation. L'interrogatoire retrouvait des ascendants saoudiens et des douleurs articulaires inflammatoires depuis l'âge de 5ans. Les signes articulaires et cutanés avaient cédé sous colchicine mais la mutation du géne MEFV n'a pas été recherchée.

## Conclusion

La maladie périodique doit être évoquée devant tout tableau simulant un érysipèle qui récidive ou persiste sous antibiothérapie adaptée.

## Conflits d’intérêts

Les auteurs ne déclarent aucun conflit d'intérêts.
